# Supplementary material for: Rad51-mediated interhomolog recombination during budding yeast meiosis is promoted by the meiotic recombination checkpoint and the conserved Pif1 helicase
Source: PLoS Genet. 2022 Dec 12;18(12):e1010407. doi: 10.1371/journal.pgen.1010407 (PMC9779700; doi:10.1371/journal.pgen.1010407)
Supplement: S3 Data — (HTML) [file pgen.1010407.s003.html]

 

 

 

 
 
 


 Tetrad Plots for hed1Δ RAD54-T132A - 

 
 
 
 
 
 
 
 
 
 
 
 

 

 
 


 


 

 

 
 


 

 


 


 

 
  Code     
 
  Show All Code  
  Hide All Code  
 
 


 Tetrad Plots for  hed1Δ RAD54-T132A 
- 

 


 
 
 
 Legend 
   
                                                                                                       
 


 

 

 

 

 
 


 
 

 
 
