# Supplementary material for: Rad51-mediated interhomolog recombination during budding yeast meiosis is promoted by the meiotic recombination checkpoint and the conserved Pif1 helicase
Source: PLoS Genet. 2022 Dec 12;18(12):e1010407. doi: 10.1371/journal.pgen.1010407 (PMC9779700; doi:10.1371/journal.pgen.1010407)
Supplement: S1 Table — (DOCX) [file pgen.1010407.s004.docx]

**S1 Table. *Saccharomyces cerevisiae* strains**

| Strain^a^ | Genotype | Source |
| --- | --- | --- |
| NH716 | *MAT***a** *leu2::hisG HIS4::LEU2-(Bam + ori)* *ho::hisG ura3(Δsma-Pst)*  *MATα* *leu2::hisG his4-X::LEU2-(NgoMIV + ori) ho::hisG ura3(ΔSma-Pst)* | [1] |
| NH942 | Same as NH716 except *hed1Δ::natMX4 dmc1Δ-2E::kanMX6* | [2] |
| NH793 | Same as NH716 except *rad51Δ::natMX4* | This work |
| NH1055 | Same as NH716 except *spo11Δ::natMX4* | [3] |
| NH2234 | Same as NH716 except  *MAT***a** *leu2::hisG HIS4::LEU2-(Bam + ori) trp1-5’Δ::hphMX4 zip1Δ::kanMX6 ndt80Δ::natMX4*  *MATα leu2::hisG his4-X::LEU2-(NgoMIV + ori) trp1-5’Δ::hphMX4 zip1Δ::kanMX6 ndt80Δ::natMX4*  *MATα leu2::hisG his4-X::LEU2-(NgoMIV + ori)* | [3] |
| NH2485 | Same as NH716 except *trp1-5’Δ::hphMX4 rrm3Δ::natMX4* | This work |
| NH2495 | Same as NH716 except *NDT80-ΔRPSKR* (*NDT80-mid*) | This work |
| NH2505 | Same as NH716 except *hed1Δ::natMX4 NDT80-mid RAD54-T132A* | This work |
| NH2528 | Same as NH716 except *hed1Δ::natMX4 RAD54-T132A* | This work |
| NH2540 | Same as NH716 except *hed1Δ::natMX4 RAD54-T132A rrm3Δ::kanMX6* | This work |
| NH2549 | Same as NH716 except *NDT80-mid rrm3Δ::kanMX6* | This work |
| NH2566 | Same as NH716 except *hed1Δ::natMX4 RAD54-T132A rad51-II3A* | This work |
| NH2596 | Same as NH716 except *hed1Δ::natMX4 RAD54-T132A NDT80-mid rrm3Δ::kanMX6* | This work |
| NH2618 | Same as NH716 except *rad51-II3A* | This work |
| NH2657 | Same as NH716 except *kanMX6*::*P_CLB2_-PIF1 (pif1-md)* | This work |
| NH2661 | Same as NH716 except *hed1Δ::natMX4 RAD54-T132A NDT80-mid pif1-md* | This work |
| NH2664 | Same as NH716 except *dmc1Δ::natMX4 trp1-5’Δ::hphMX4* | ­­­This work |
| NH2670 | Same as NH716 except *hed1Δ::natMX4 RAD54-T132A NDT80-mid rrm3Δ::hphMX4 pif1-md* | This work |
| NH2671 | Same as NH716 except *trp1-5’Δ::hphMX4 rrm3Δ::kanMX6 pif1-md* | This work |
| NH2693 | Same as NH716 except  *hed1Δ::natMX4 RAD54-T132A dmc1Δ::hphMX4*  *kanMX6::P_CLB2_-3HA-PIF1* | This work |
| NH2701 | Same as NH716 except *dmc1Δ::hphMX4*  *hed1Δ::natMX4 RAD54-T132A* | This work |
| NH2704 | Same as NH716 except *hed1Δ::natMX4 RAD54-T132A rrm3Δ::hphMX4 pif1-md* | This work |
| NH2714 | Same as NH716 except  *hed1Δ::natMX4 RAD54-T132A dmc1-II3A* | This work |
| NH2716 | Same as NH716 except *dmc1Δ::hphMX4*  *hed1Δ::natMX4 kanMX6::P_CLB2_-3HA-PIF1* | This work |
| NH2725 | Same as NH716 except *NDT80-mid pif1-md* | This work |
| NH2691 | Same as NH716 except *hed1Δ::natMX4 RAD54-T132A pif1-md* | This work |
| NH2598 RCEN | Same as NH716 except *HIS4::leu2ΔI::kanMX6-(Bam+ ori) TRP1 CEN8::RFP::LEU2*  *his4-X::LEU2-(NgoMIV + ori) trp1-5’Δ::hphMX4 CEN8::CFP::TRP1* | This work |
| NH2610 RCEN | Same as NH2598 RCEN except *hed1Δ::natMX4 NDT80-mid RAD54-T132A* | This work |
| NH2616 RCEN | Same as NH2598 RCEN except *hed1Δ::natMX4 RAD54-T132A* | This work |
| NH2623 RCEN | Same as NH2598 RCEN except *trp1-5’Δ::hphMX4 rrm3Δ::natMX4* | This work |
| NH2629 RCEN | Same as NH2598 RCEN except *hed1Δ::natMX4 RAD54-T132A rrm3Δ::kanMX6* | This work |
| NH2634 RCEN | Same as NH2598 RCEN except *NDT80-mid* | This work |
| NH2637 RCEN | Same as NH2598 RCEN except *hed1Δ::natMX4 RAD54-T132A NDT80-mid rrm3Δ::kanMX6* | This work |
| NH2666 RCEN | Same as NH2598 RCEN except *rad51-II3A* | This work |
| NH2685 RCEN | Same as NH2598 RCEN except *P_CLB2_-PIF1 (pif1-md)* | This work |
| NH2687 RCEN | Same as NH2598 RCEN except *hed1Δ::natMX4 NDT80-mid RAD54-T132A pif1-md* | This work |
| NH2702 RCEN | Same as NH2598 RCEN except *hed1Δ::natMX4 RAD54-T132A pif1-md* | This work |
| NH2705 RCEN | Same as NH2598 RCEN except *hed1Δ::natMX4 NDT80-mid RAD54-T132A rad51-II3A* | This work |
| NH2741 RCEN | Same as NH2598 RCEN except *hed1Δ::natMX4 NDT80-mid RAD54-T132A pif1-md rad51-II3A* | This work |
| NH2598 RGC | Same as NH716 except *HIS4::leu2ΔI::kanMX6-(Bam+ ori) TRP1*  *his4-X::LEU2-(NgoMIV + ori) trp1-5’Δ::hphMX4*  *CEN8::RFP::LEU2 ARG4* *THR1*  *CEN8*  *ARG4::GFP*-URA3 THR1::CFP::TRP1* | This work |
| NH2610 RGC | Same as NH2598 RGC except *hed1Δ::natMX4 NDT80-ΔRPSKR RAD54-T132A* | This work |
| NH2616 RGC | Same as NH2598 RGC except *hed1Δ::natMX4 RAD54-T132A* | This work |
| NH2634 RGC | Same as NH2598 RGC except *NDT80-mid* | This work |
| NH2687 RGC | Same as NH2598 RGC except *hed1Δ::natMX4 RAD54-T132A NDT80-mid* *pif1-md* | This work |
| NH2695 RGC | Same as NH2598 RGC except *pif1-md* | This work |
| NH2702 RGC | Same as NH2598 RGC except *hed1Δ::natMX4 RAD54-T132A pif1-md* | This work |
| NH2233 | *MAT***a** *leu2ΔhisG his4-x ARG4 ura3 lys2 ho::LYS2 ndt80Δ::hphMX4 red1Δ::kanMX6*  *MATα leu2-k HIS4 arg4-Nsp ura3 lys2 ho::LYS2 ndt80Δ::hphMX4 red1Δ::kanMX6* | This work |
| NH2234 | Same as NH2179 except *ndt80Δ::natMX4* | [3] |
| NH2694-a2 | *MAT***a** *his4::URA3rev-tel-ARG4+SNPs RNQ1::hphMX::FUS1 ura3Δ arg4Δ hed1Δ::NS RAD54-T132A msh2Δ::kanMX6* | This work |
| NH2695-f8 | *MATα leu2-R STE50::natMX::RRP7 his4::URA3rev-tel-ARG4 ura3Δ arg4Δ hed1Δ::NS*  *RAD54-T132A msh2Δ::kanMX6* | This work |
| NH2700 | *MAT***a** *LEU2 STE50 RRP7 his4::URA3rev-tel-ARG4+SNPs RNQ1::hphMX::FUS1*  *MATα leu2-R STE50::natMX::RRP7 his4::URA3rev-tel-ARG4 RNQ1 FUS1*    *ura3Δ arg4Δ hed1Δ::NS RAD54-T132A msh2Δ::kanMX6*  *ura3Δ arg4Δ hed1Δ::NS RAD54-T132A msh2Δ::kanMX6* | This work |

**REFERENCES**

1. Callender TL, Hollingsworth NM. Mek1 suppression of meiotic double-strand break repair is specific to sister chromatids, chromosome autonomous and independent of Rec8 cohesin complexes. Genetics. 2010;185(3):771-82.

2. Liu Y, Gaines WA, Callender T, Busygina V, Oke A, Sung P, et al. Down-regulation of Rad51 activity during meiosis in yeast prevents competition with Dmc1 for repair of double-strand breaks. PLoS Genet. 2014;10(1):e1004005.

3. Chen X, Suhandynata RT, Sandhu R, Rockmill B, Mohibullah N, Niu H, et al. Phosphorylation of the synaptonemal complex protein Zip1 regulates the crossover/noncrossover decision during yeast meiosis. PLoS Biol. 2015;13(12):e1002329.
