# Supplementary material for: Rad51-mediated interhomolog recombination during budding yeast meiosis is promoted by the meiotic recombination checkpoint and the conserved Pif1 helicase
Source: PLoS Genet. 2022 Dec 12;18(12):e1010407. doi: 10.1371/journal.pgen.1010407 (PMC9779700; doi:10.1371/journal.pgen.1010407)
Supplement: S2 Table — (DOCX) [file pgen.1010407.s005.docx]

**S2 Table. Plasmids**

| **Plasmid name** | **Relevant yeast genotype** | **Source** |
| --- | --- | --- |
| pRS304 | *TRP1* | [1] |
| pRS306 | *URA3* | [1] |
| YIp5 | *URA3* | [1] |
| pMJ787 | *kanMX6::P_CLB2_* | Michael Lichten |
| pFA6a-kanMX6 | *kanMX6* | [2] |
| p4339 | *natMX4* | [3] |
| pAG32 | *hphMX4* | [3] |
| pNH257 | *URA3 P_REC8_* | This work |
| pNH317 | *URA3 NDT80-mid* | [4] |
| pHN104 | *URA3 RAD54-T132A* | [5] |
| pSZ5 | *LEU2 CAS9-gDNA-natMX4* | Bruce Futcher |
| pBG22 | *URA3 RRM3* | This work |
| pJW5 | *URA3 PIF1* | This work |
| pJW5-m1 | *URA3 pif1-m1* (*m1* = M1A) | This work |
| pJW7 | *URA3 P_REC8_-RRM3* | This work |
| pJW11 | *URA3 PIF1-3FLAG* | This work |
| pJW14 | *URA3 pif1-m1-3FLAG* | This work |
| pJW14-K264A | *URA3 pif1-m1-K264A-3FLAG* | This work |
| pJW14-R3E | *URA3 pif1-m1-R3E-3FLAG (R3E=* I817R, M820R, L821R, R823E*)* | This work |
| pAM1-II3A | *URA3 rad51-II3A* (*II3A =* R188A K361A K371A) | This Work |
| pSK693 | *LEU2 pYKL050c-RFP* (targets integration near *CEN8*) | [6] |
| pSK694 | *TRP1 pYKL050c-CFP* (targets integration near *CEN8*) | [6] |
| pSK695 | *TRP1 pYKL050c-CFP* (targets integration near *THR1*) | [6] |
| pSK729 | *URA3 pYKL050c-GFP** (targets integration near *ARG4*) | [6] |
| pRD1 | *URA3 dmc1-II3A* (R124A R298A K308A) | This work |

**REFERENCES**

1. Parent SA, Fenimore CM, Bostian KA. Vector systems for the expression, analysis and cloning of DNA sequences in *S. cerevisiae*. Yeast. 1985;1(2):83-138. doi: 10.1002/yea.320010202.

2. Longtine MS, McKenzie A, 3rd, Demarini DJ, Shah NG, Wach A, Brachat A, et al. Additional modules for versatile and economical PCR-based gene deletion and modification in *Saccharomyces cerevisiae*. Yeast. 1998;14(10):953-61.

3. Goldstein AL, McCusker JH. Three new dominant drug resistance cassettes for gene disruption in *Saccharomyces cerevisiae*. Yeast. 1999;15(14):1541-53.

4. Chen X, Gaglione R, Leong T, Bednor L, de Los Santos T, Luk E, et al. Mek1 coordinates meiotic progression with DNA break repair by directly phosphorylating and inhibiting the yeast pachytene exit regulator Ndt80. PLoS Genet. 2018;14(11):e1007832. Epub 2018/11/30. doi: 10.1371/journal.pgen.1007832.

5. Niu H, Wan L, Busygina V, Kwon Y, Allen JA, Li X, et al. Regulation of meiotic recombination via Mek1-mediated Rad54 phosphorylation. Mol Cell. 2009;36(3):393-404. doi: 10.1016/j.molcel.2009.09.029.

6. Thacker D, Lam I, Knop M, Keeney S. Exploiting spore-autonomous fluorescent protein expression to quantify meiotic chromosome behaviors in *Saccharomyces cerevisiae*. Genetics. 2011;189(2):423-39. Epub 2011/08/16. doi: 10.1534/genetics.111.131326.
