# Supplementary figures and images for: Rad51-mediated interhomolog recombination during budding yeast meiosis is promoted by the meiotic recombination checkpoint and the conserved Pif1 helicase
Source: PLoS Genet. 2022 Dec 12;18(12):e1010407. doi: 10.1371/journal.pgen.1010407 (PMC9779700; doi:10.1371/journal.pgen.1010407)

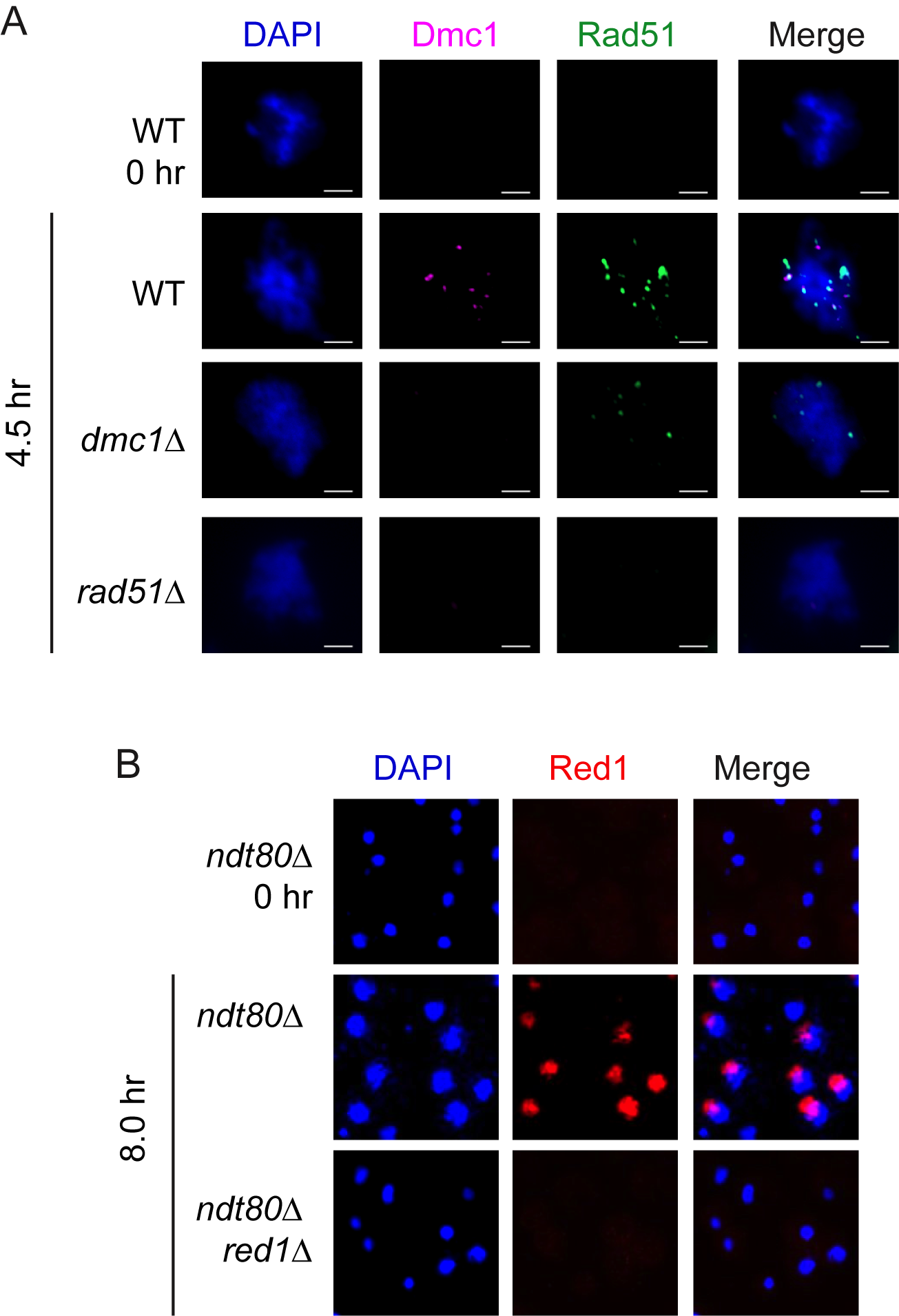

Supplement: S1 Fig — (A) Detection of Dmc1 and Rad51 foci. Chromosome spreads at the indicated timepoints in Spo medium made from WT (NH716), dmc1Δ (NH2664), or rad51Δ (NH793) were probed with antibodies against either Rad51 or Dmc1 and detected by indirect immunofluorescence. DAPI staining was used to detect DNA. Scale bar is 2 μm. (B) Whole cell Red1 immunofluorescence. The diploids ndt80Δ (NH2234) and ndt80Δ red1Δ (NH2233) were incubated in Spo medium for the indicated times. Cells were fixed, stained with DAPI and probed with α-Red1 antibodies. Red1 was detected by indirect immunofluorescence. (TIF) [file pgen.1010407.s006.tif]

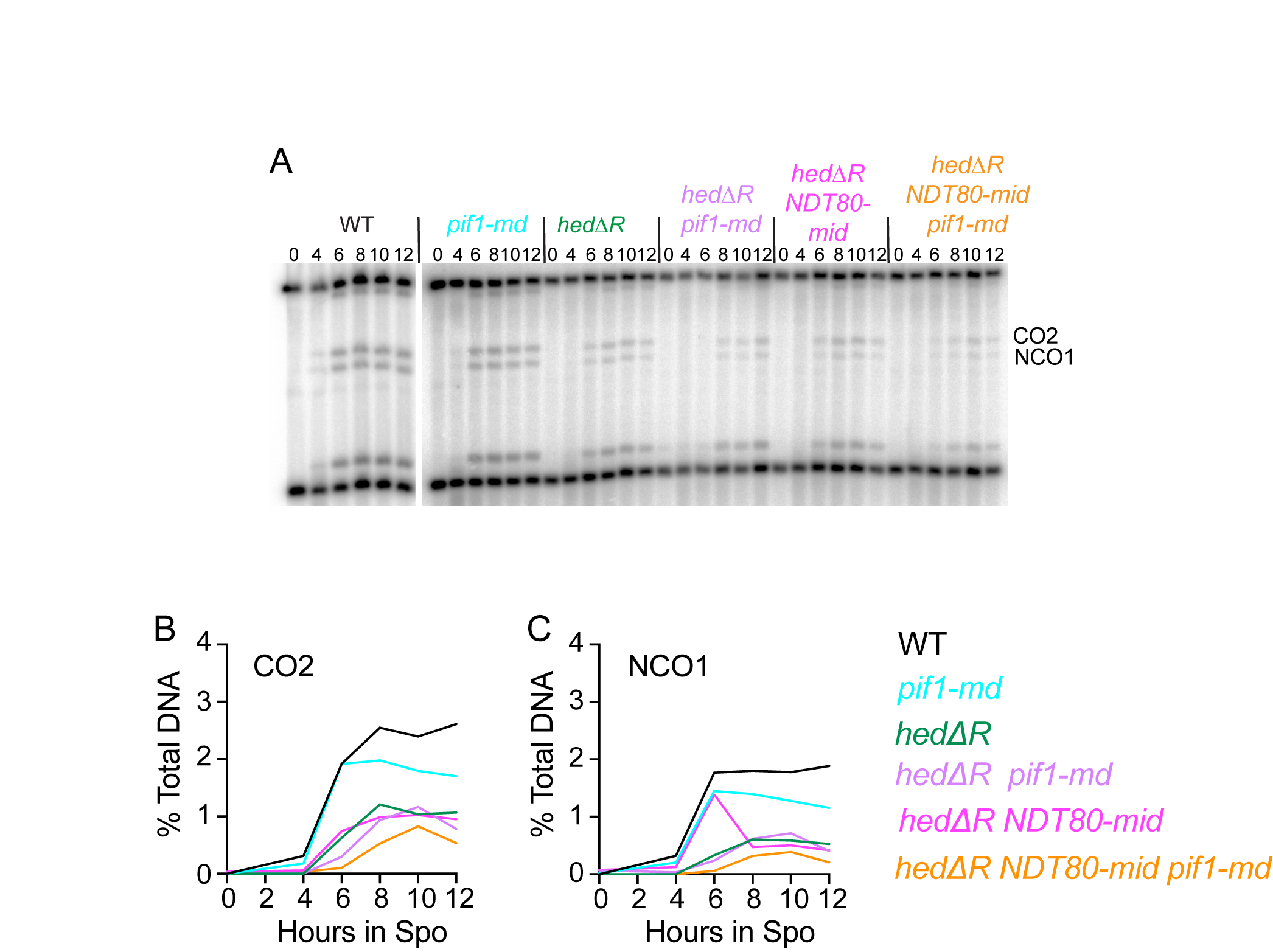

Supplement: S2 Fig — (A) Southern blot of one of two timecourses used for this Figure containing WT, hedΔR, hedΔR NDT80-mid, pif1-md (NH2657), hedΔR pif1-md (NH2691) and hedΔR NDT80-mid pif1-md (NH2661). Genomic DNA was digested with XhoI and NgoMIV to detect the CO2 and NCO1-specific bands described in Fig 4A. (B) Quantification of CO2 showing the average values from two different timecourses. (C) Quantification of NCO1 showing the average values from two different timecourses. Note that one of the hedΔR NDT80-mid replicates exhibited an unusually high value at the six hour timepoint which skewed the result. (TIF) [file pgen.1010407.s007.tif]

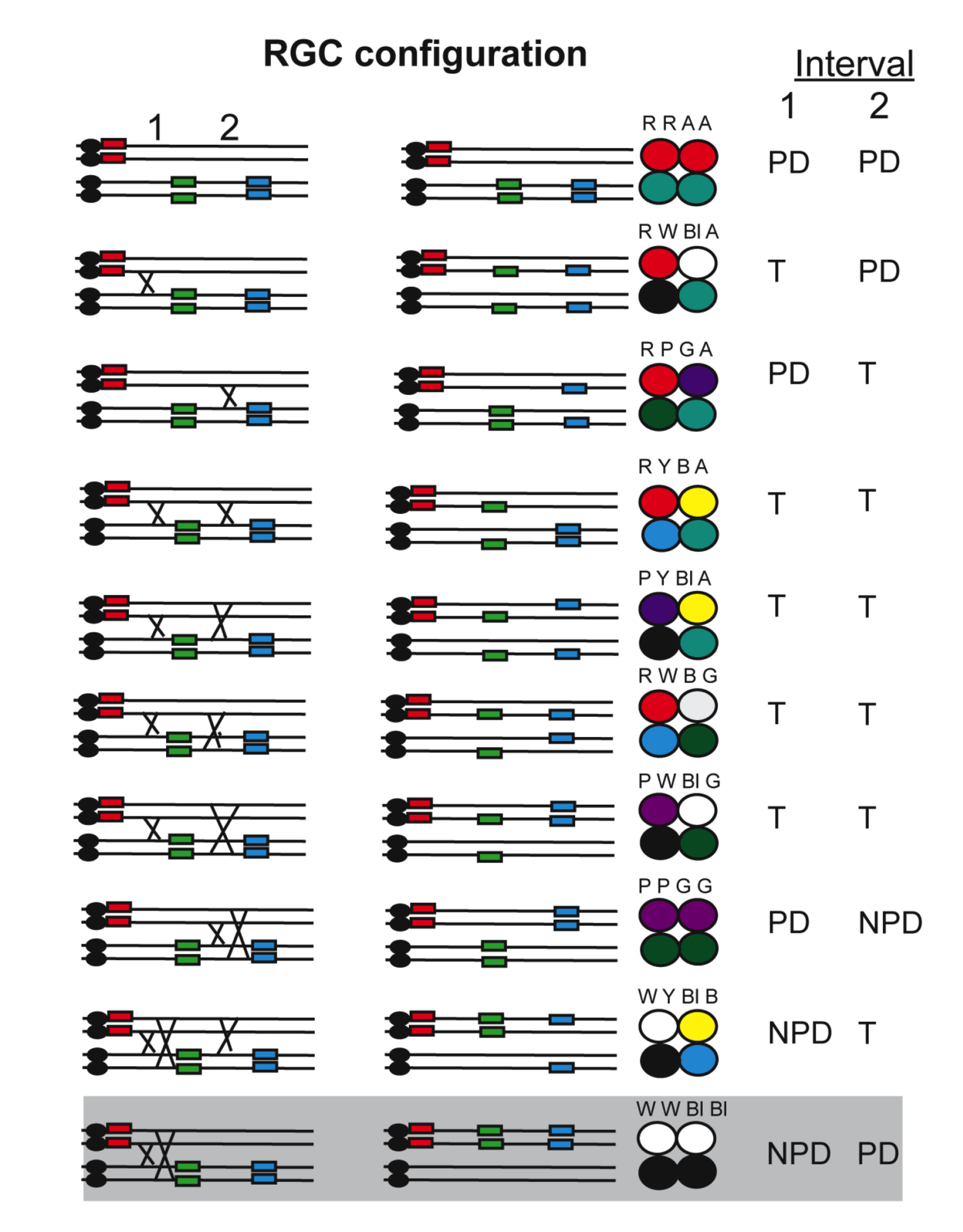

Supplement: S3 Fig — The left side shows the positions of crossovers (indicated by an “X”) between CEN8::RFP and ARG4::GFP* (Interval 1) and ARG4::GFP* and THR1::CFP (Interval 2). The next column shows the configuration of fluorescent protein genes on each chromatid after the indicated crossovers. The table indicates whether a given interval is a parental ditype (PD), tetratype (T) or nonparental ditype (NPD). The tetrad indicated in the gray shaded part of the diagram can be due either to an NPD in Interval 1 or MI nondisjunction. R = red, A = aqua, W = white, Bl = black, P = purple, G = green, Y = yellow, B = blue. (TIF) [file pgen.1010407.s008.tif]

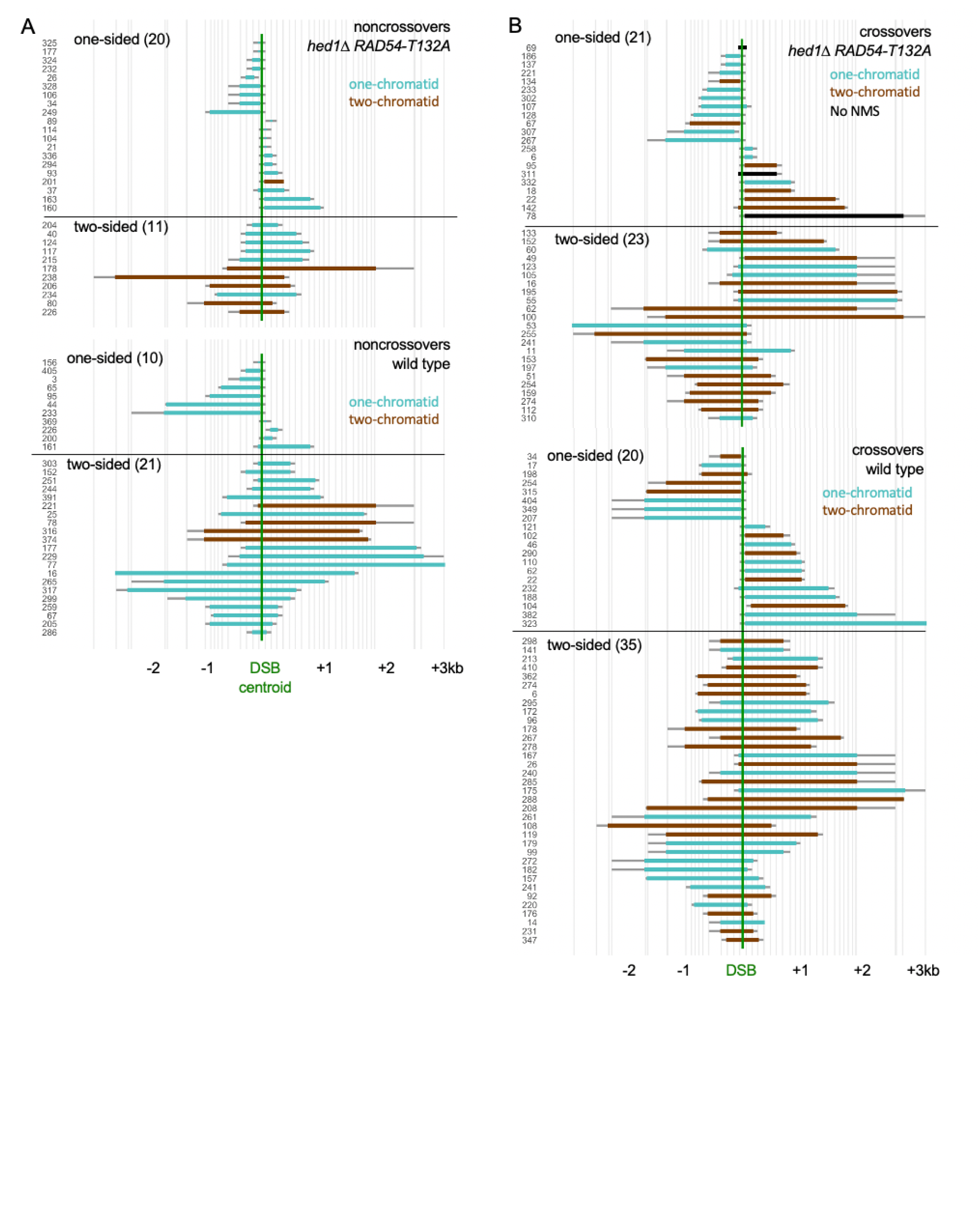

Supplement: S4 Fig — Gene conversion tracts in noncrossovers (A) and crossovers (B) in hedΔR (top) and wild type (bottom). Thick colored bars and thin gray bars indicate minimum and maximum NMS tracts, respectively. Turquoise—heteroduplex on one chromatid; brown—heteroduplex on two chromatids. Vertical axis–tetrad identifiers; vertical lines—marker positions. Underlying data are in S2 Data, Sheet 6 and in [127]. (TIF) [file pgen.1010407.s009.tif]

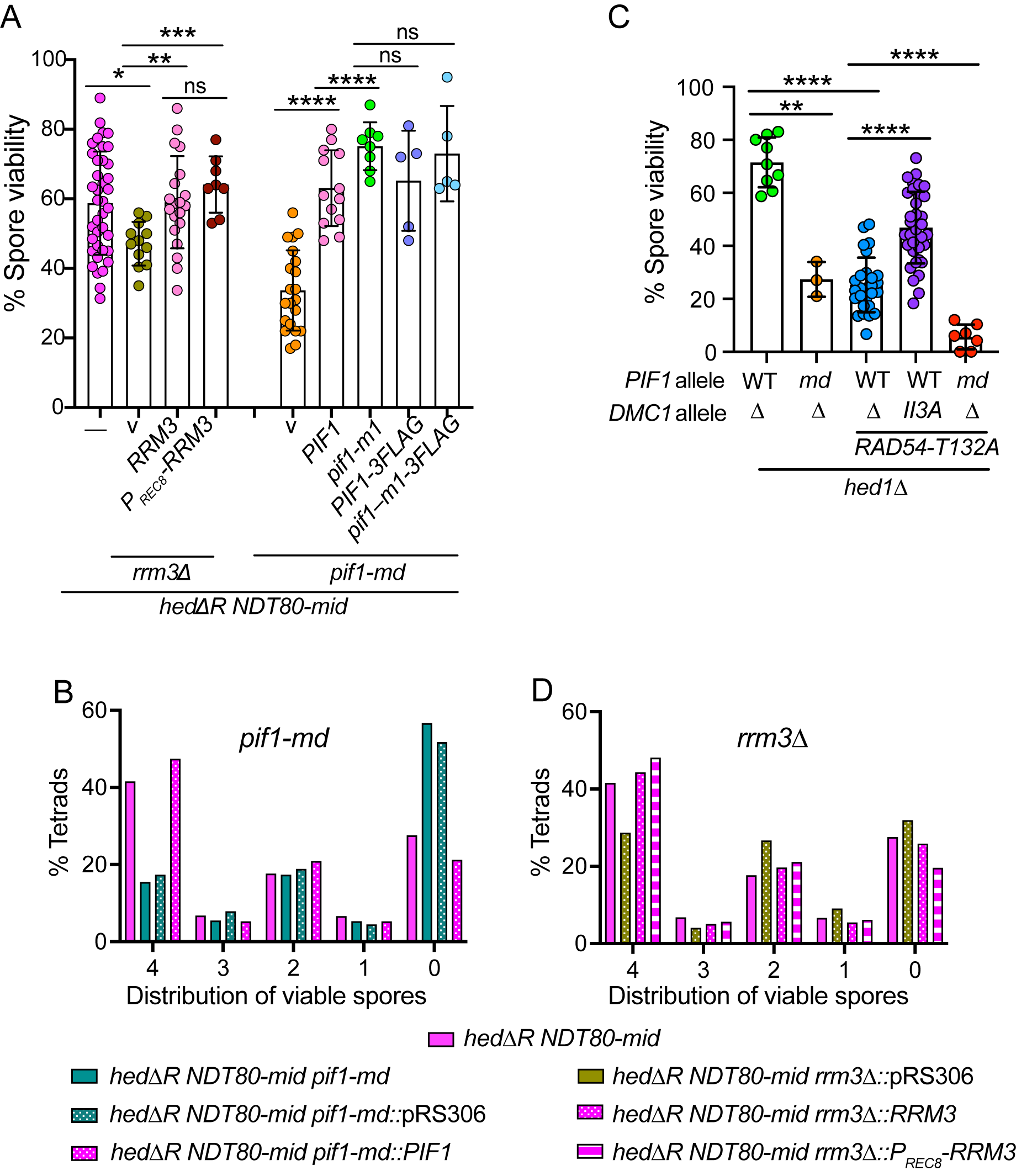

Supplement: S5 Fig — (A) Spore viability. Single colonies of the hedΔR NDT80-mid rrm3Δ diploid (NH2596) containing two copies of the vector (v) (pRS306), RRM3 (pBG22) or PREC8-RRM3 (pJW7) and the hedΔR NDT80-mid pif1-md diploid, NH2661, containing two copies of pRS306, PIF1 (pJW5), pif1-m1 (pJW5-m1), PIF1-3FLAG (pJW11) or pif1-m1-3FLAG (pJW14) were sporulated on solid medium and at least 20 tetrads were dissected per transformant. The statistical significance of differences between strains was determined using the Mann-Whitney test (* = p < .05; ** = p < .01; *** = p < .001, **** = p < .0001). (B) Distribution of viable spores in tetrads for a subset of the hedΔR NDT80-mid pif1-md dissections shown in Panel A. (C) Single colonies of hed1Δ dmc1Δ (NH942), hed1Δ dmc1Δ pif1-md (NH2716), hedΔR dmc1Δ (NH2701), hedΔR dmc1-II3A (NH2714), and hedΔR dmc1Δ pif1-md (NH2693) were sporulated and dissected and analyzed as described for Panel A. (D) Distribution of viable spores in tetrads for the hedΔR NDT80-mid rrm3Δ dissections shown in Panel A. (TIF) [file pgen.1010407.s010.tif]

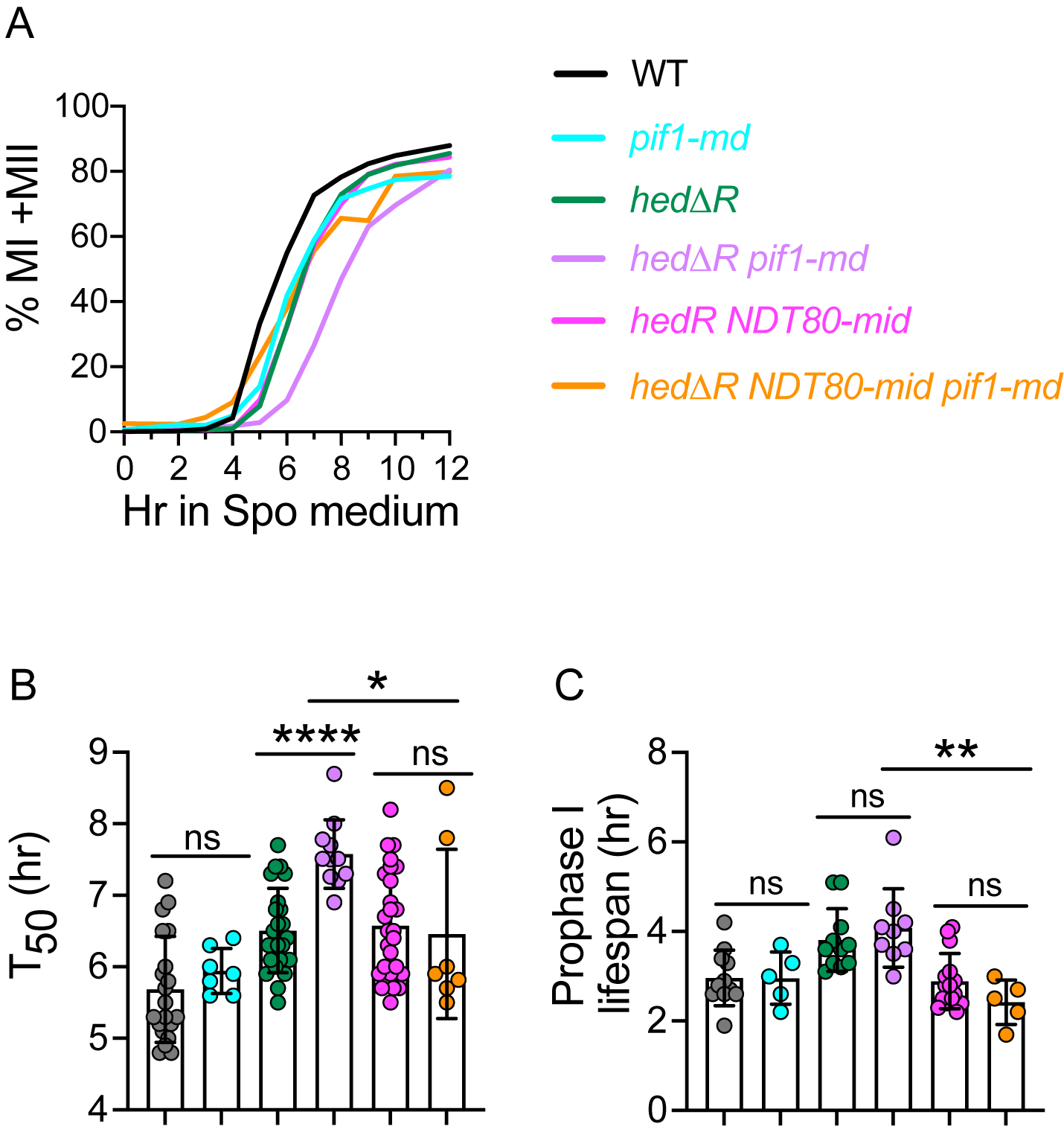

Supplement: S6 Fig — (A) Meiotic progression. Timecourse analysis to determine the percentage of bi and tetranucleate cells was performed as in Fig 1A and the average %MI + MII values plotted for each timepoint. The strains and data for WT, hedΔR, and hedΔR NDT80-mid are the same as in Fig 3A with the addition of two biological replicates. The new strains were pif1-md (NH2657) (n = 7), hedΔR pif1-md (NH2691) (n = 11) and hedΔR NDT80-mid pif1-md (NH2661) (n = 7). (B) T50 values calculated for the timecourses in Panel A. (C) Prophase lifespan. The data for WT, hedΔR and hedΔR NDT80-mid for Panels B and C were taken from Fig 3. Data for pif1-md containing strains is from the timecourses in Panel A. (TIF) [file pgen.1010407.s011.tif]
